# Barriers to and facilitators of care for hemodialysis patients; a qualitative study

**DOI:** 10.15171/jrip.2016.09

**Published:** 2016-02-28

**Authors:** Monir Nobahar, Mohammad Reza Tamadon

**Affiliations:** ^1^Nursing Care Research Center, Faculty of Nursing and Paramedical, Semnan University of Medical Sciences, Semnan, Iran; ^2^Department of Internal Medicine, Faculty of Medicine, Semnan University of Medical Sciences, Semnan, Iran

**Keywords:** Barriers, Facilitators, Caring, Patients, Nursing, Hemodialysis, Qualitative research

## Abstract

**Introduction:** Patients undergoing hemodialysis require direct and continuous care. Identifying the barriers to and factors facilitating hemodialysis care can improve care quality.

**Objectives:** The aim of this study was to assess the barriers and facilitators of care for hemodialysis patients.

**Patients and Methods:** This study was conducted as a qualitative study and it utilized content analysis approach. The study was performed in hemodialysis ward of Kowsar hospital in Semnan, in 2014. We used purposive sampling method with maximum diversity. Semi-structured interviews with open questions were used to collect data from a total of 20 participants.

**Results:** The main topic of health care challenges was divided into two main categories, including the facilitators and barriers of hemodialysis care. The facilitators of hemodialysis care had four subcategories, including "intimate relationship", "basic knowledge", "hemodialysis skills", and "experience". The category of barriers had eight subcategories, including "shortage of nurses and heavy workload", "weak authority of the head nurse", "ignorant director of nursing", "shortage of nephrologists", "lack of vascular surgery expert", "lack of nurse’s aide and nursing assistant ", "unskilled staffs", and "interference by patients’ caregivers".

**Conclusion:** The findings of this study showed that access to human resources and their abilities were among the factors facilitating care. However, lack of qualified medical staff at each level of care delivery was one of the barriers to hemodialysis care. Hence, it is of great importance for policy makers, managers, and program designers to recruit human resources who have the characteristics and competencies required for providing hemodialysis care.

**Implication for health policy/practice/research/medical education:**

In a study on a group of hemodialysis patients, in Semnan of Iran, we found that, access to human resources and their abilities were among the factors facilitating care. However, lack of qualified medical staff at each level of care delivery was one of the barriers to hemodialysis care. Hence, it is of great importance for policy makers, managers, and program designers to recruit human resources who have the characteristics and competencies required for providing hemodialysis care.

## Introduction

Chronic kidney disease is a growing health problem across the world (1). Patients with chronic renal failure are in need of renal replacement therapy and they should be treated with hemodialysis. Both chronic kidney disease and hemodialysis cause many problems and needs in the patients, which often cannot be fulfilled by patients. In addition, the complications of the disease and hemodialysis treatment, both are among the fundamental causes of frequent hospitalization, poor quality of life, and heavy burden of care (2). Delivery of care for these patients, before and after the initiation of dialysis, is less than optimal level, resulting in a high financial burden and unfavorable clinical outcomes for patients and the health care system (1). Delivery of continuous and coordinated care reduces the demand for hospital services, waiting times for other services in different parts of the hospital, and the risk of complications (2). Patients and their families are always in need of care, guidance, training, and ongoing support provided by the health team (3,4). According to Tejada-Tayabas et al (2), one of the main barriers to the delivery of care for hemodialysis patients is the heavy workload in hemodialysis wards; the heavy workload is caused due to different factors, including; need to provide several services, lack of nurses and other professionals, involvement of families and caregivers in the process of care delivery, lack of a strategy for systematic training and guidance for the patients, families, and caregivers, and some patients’ families ignorance toward their patients conditions (2). According to the study of Griva et al (5), the main barriers to the delivery of care in hemodialysis patients include the followings items; forgetfulness, safety concerns, lack of understanding and knowledge, poor communication, and lack of control over social pressures. They also introduced two groups of factors facilitating hemodialysis care, including internal factors (self-starting factors) and external factors (started by parents, professionals, other professional members of the health care team, and other hemodialysis patients) (5). Chenitz et al (6) found that the explanations presented by care team members and also the relationship with other dialysis patients are among the facilitators of dialysis care. They believe that the identification of barriers and facilitating factors of hemodialysis can help to design special recommendations for this special group of patients; it can also increase their desire to continue hemodialysis treatment (6). Because of various diseases and physical and psychological problems, this group of patients is in need of continuous and comprehensive patient care to ensure their adherence to therapy. However this type of care is seldom provided for patients (2). In many dialysis centers, the delivery of health care services by health care providers is very complex and people who are affected by the problems have different levels of sensitivity to the treatment. However, the existing evidences suggest that, using a promising approach, these conditions can be appropriately managed and the outcomes of intervention could be improved for this vulnerable population and the obstacle would be overcame (1).

## Objectives

Quantitative studies have mainly focused on quality of life and the needs of these patients (3) while qualitative research is needed to describe the experience of patients (7). Qualitative research can evaluate people’s experiences of living with the disease, interpret their life experiences, and show the effects of the disease on their lives (8,9). Hence, the aim of this study was to evaluate the barriers and facilitators of care in hemodialysis patients.

## Patients and Methods

In this study we used conventional content analysis method. Conventional content analysis method is an appropriate method to obtain valid and reliable results out of textual data; the results can be used to reach and develop knowledge, new insights, facts, and a practical guide to performance (10). This study was carried out in hemodialysis ward. The studied population included hemodialysis patients, caregivers, nurses, and physicians. Participants were selected via purposive sampling. Sampling continued until data saturation (11). A total of 20 participants, including 8 patients, 7 nurses, 3 caregivers, and 2 physicians participated in this study.

Data was collected through semi-structured interviews, field notes, and hand notes. The interview questions were about the nursing care provided for hemodialysis patients. Interviews began with a general open question: “Please talk about nursing care”. Based on the derived categories, the following interview questions were formulated. To encourage the participants and to achieve more deep information, we used probe questions like “can you explain more?” or “give an example”. Each interviews lasted 40 to 85 minutes. The interviews were carried out in a room at the clinical part of the Hospital, next to the hemodialysis ward. At the time of the interviews, only researchers and participants were present in the room.

### Statistical analysis

Data analysis was performed based on the five-steps suggested by Granheim and Lundman which included:

1) Transcribing the entire interview immediately after each interview,
2) Reading the whole transcript to reach a general understanding of its contents,
3) Identifying the units of meaning and the primary codes,
4) Categorizing the similar primary codes and putting them in a more comprehensive category,
5) Determining the latent content (10).

We used Lincoln and Guba criteria to ensure the rigor of the collected data. The credibility of the collected data was confirmed via prolonged engagement, checking the content of interviews, and analysis of the codes via member check. Dependability of the findings was ensured via prescribing the text at the earliest time possible and also via external check i.e. taking the comments of colleagues about the process of conducting interviews, analysis, data mining, and review of all the data. When collecting the data, we used time triangulation method and selected the participants with maximum variation so that to increase data reliability and data confirmability. Furthermore, the transcribed content, codes, and extracted categories were checked by two faculty members (peer check). Transferability or fittingness of data was approved via providing direct quotes and examples, rich explanation of the data, and scientific consultations with experts (12).

### Ethical issues

1) The research followed the tenets of the Declaration of Helsinki; 2) All participants singed an informed consent from to participate in the study. To observe the ethical issues, we followed the principle of confidentiality and did not reveal the information and the name of the participants. In addition, participants were allowed to withdraw from the research any time they wished; and 3) the research was approved by the ethical committee of Semnan University of Medical Sciences.

## Results

A total 20 participants were studied (Table 1).

**Table 1:** Demographic characteristics of participants

**Participants**	**Min-Max**	**Mean**
Age (years)		
Patients	35-75	51.37
Nurses	37-60	46.5
Physicians	45-50	47.5
Caregivers	24-84	50.73
Gender		
Patients	5 women - 3 men	
Nurses	5 women - 2 men	
Physicians	1 women - 1 men	
Caregivers	3 men	
Education		
Patients	6 primary education, 2 high school diploma.	
Nurses	Bachelor of nursing	
Physicians	1 proficient, 1 specialist	
Caregivers	1 primary education, 2 high school diploma	
Job		
Patients	2 unemployed, 1 retired, all women were housewives	
Nurses	Nurse, head nurse	
Physicians	Doctor	
Caregivers	2 retired, 1 worker	
History of dialysis	3 to 21 years	9.37 years
Marital status		
Patients	6 married, 1 singles, 1 spouse died	
Nurses	4 married, 2 singles, 1 divorced	
Physicians	2 married	
Caregivers	2 married, 1 singles	
Number of children		
Patients	2-8	3.25
Nurses	1-2	1.8
Physicians	2	2
Caregivers	4-8	4

### Challenges in the delivery of care for hemodialysis patients

The main theme was the challenges of care. Delivery of care for a long time was faced with several challenges. These themes included two general categories of barriers to and facilitators of care.

### Facilitators of care

The participants put a great emphasis on the characteristics and competencies of nurses which are required to facilitate the delivery of care for the patients. This category includes four subcategories of “intimate relationship”, “basic knowledge”, “hemodialysis skills”, and “experience”.

### Intimate relationship

Patients expected an intimate, friendly, and kind relationship with the nurses; they also expected nurses to be good-tempered, respect the patients, and observe the rules of hemodialysis ward.

“*When a nurse treats me bad, speak to me in a very ugly and nasty tone and tell me “you are only a patient and I am a nurse, who knows better, you or me? You should be quiet”. Nurse must be a little bit kinder and more well-mannered*” (Patient No. 5).

### Basic knowledge

Nurses must receive specialized training about the use of dialysis devises and how to control the complications of hemodialysis.

“*The nurse should have enough information about kidney and electrolytes, because a small error in the selection of filter, blood flow, and UF could lead to the worst electrolytic problem*” (Nurse No. 6).

### Obtaining hemodialysis skills

Only those nurses can properly provide care for patients who are able to use the needles easily, have skills to work with profiling system of a hemodialysis machine, etc.

“*First of all, a nurse must be able to fully access the vessels, because 50% of dialysis process depends on the nurses’ skills to access the vessels. In addition, it is of great importance to match the patient with a filter or hemodialysis device*” (Nurse No. 1).

### Experience

Because of the absence of nephrologists, residents, and even interns in all the shifts of dialysis, the nurses must have sufficient clinical experience, to gain patients’ trust and ensure a successful dialysis.

“*The books recommend us to be careful of air embolism. However, we do not have any idea of this problem and we do not know what to do until we face it by ourselves*” (Nurse No. 4).

### Barriers to care for hemodialysis patients

The barriers can lead to a reduction in the quality of care for patients undergoing hemodialysis. The category of barriers had eight subcategories, including “shortage of nurses and heavy workload”, “weak authority of the head nurse”, “ignorant director of nursing”, “shortage of nephrologists”, “lack of vascular surgery expert”, “lack of nurse’s aide and nursing assistant”, “unskilled staffs”, and “interference by patients caregivers”.

### Shortage of nurses and heavy workload

There must be sufficient nursing personnel in hemodialysis ward so that they provide care services on time to meet the needs of patients.

“*A nurse is just one person but he must provide dialysis services for eight patients. Her work is so hard. What should he do?”* (Patient No. 6).

### Weak authority of the head nurse

The head nurse must supervise the work of nurses, be aware of the patients’ conditions, and, if necessary, help the nurse to provide care for the patients.

“*I have never seen a head nurse visit a patient and check his condition. He never check the results of the laboratory tests and ask the patient why he has eaten something which changed the results of the tests*” (Patient No. 7).

### Ignorant director of nursing

The director must use the best nurses as the head nurse and use the most qualified nurses to deliver care services for hemodialysis patients.

“*She doesn’t overlooks the efficiently, knowledge, and incentives of nurses who are invited to work in dialysis ward. It is not good at all, because it is harmful for the director, the patients, colleagues, and even for the government*” (Nurse No. 6).

### Shortage of nephrologists

The participants put emphasis on the need for the presence of nephrologist in hemodialysis ward so that they provide patients with the necessary visits and advices.

*“A specialized physician is needed for the ward to handle and solve our problems”* (Patient No. 3).

### Lack of vascular surgery experts

The lack of vascular surgery experts has led to many problems for patients (inappropriate re-catheterization with a high cost) and nurses (repeated attempts and the inability to access sufficient blood).

*“When a patient needs a subclavian catheter, physicians try to avoid this process and ask other physicians to do that. As a result, the patient must visit a private office to install the catheter and he spends a lot of money. It may work or not”* (Nurse No. 7).

### Lack of nurse’s aide and nursing assistant

To provide optimal care for patients, it is necessary to have a nursing team in the hemodialysis ward, however according to the participants, there are only some nurses, and crews in the hemodialysis ward.

*“The organizational chart did not specify any place for nursing assistants. The presence of two nursing assistant can help too much, as they can do much of the initial works for the patients”* (Nurse No. 3).

### Unskilled staffs

These staffs are not stable and do not receive enough training; in addition, some of the direct services for hemodialysis patients are provided by this group of staffs. As a result of such conditions, some problems have emerged.

*“We work with dialysis solution, Prime container, and disinfectant solution, and the service staffs should recognize all of them. Once we observed some problems with the conduct device, later we found that one of the staffs had mixed the prime with the other solutions*” (Nurse No. 2).

### Interference by patients’ caregivers

The presence of caregivers in hemodialysis ward, in many cases, led to some problems in the process of delivery of care to the patients and increases the risk of infection.

“*Some caregivers are willing to disrupt the nursing activities. They even give comments about the filters used for the patients. They tell the nurses not to use a specific filter, or they recommend a specific conductor*” (Nurse No. 4).

## Discussion

The results of this study showed that to facilitate hemodialysis care it is necessary to recruit efficient human resources and nurses who would be able to establish close relationships with patients, have basic knowledge, be able to achieve hemodialysis skills, and have enough experience. On the other hand, the shortage of nurses, heavy workload, weak authority of the head nurse, ignorant director of nursing, shortage of nephrologists, lack of vascular surgeon, lack of nurse’s aide and nursing assistant, unskilled staff, and interventions by caregivers are among the barriers to hemodialysis care. The results of this study are in line with the findings of Walker et al study (13); they also believe that the delivery of hemodialysis care requires special technical abilities; they also say that providing care services for hemodialysis patients is a challenge which is associated with increased supervisory activities and needs many human resources and equipments to provide different services including emergency medical consultations, surgery, internal specialists, and intensive care (13). Guidelines, trainings, and ongoing support from treatment team must be provided for patients and their families so that they can easily deal with and accept changes in their lifestyle (14). Such services can increase patients’ life expectancy and even can provide support for caregivers (15).

According to the results of this study, the subcategories of intimate communication, basic knowledge, access to hemodialysis skills, and experience of nurses were among the factors facilitating hemodialysis care. Rajeswari found that the nurses are directly responsible for providing care for patients undergoing hemodialysis (16). To provide high quality nursing care, the nurses may take advantage of their technical knowledge, clinical skills, and interpersonal relationships (intimacy, humor, and training patient) (17).

According to the results of this study, the intimate relationship between nurses and patients could facilitate the delivery of hemodialysis care for the patients. Clinical nurses who spend time with patients can provide continuous care and improve communication between hemodialysis team members (18). The most important behavior during hemodialysis which patients expect from their nurses in an ethical and proper behavior (19).

The participants in this study believed that basic knowledge about hemodialysis was one of the factors facilitating care services for patients. According to Bennett study, nurses believe that the basic knowledge has a significant impact on the quality of nursing care (17). The patients’ and health care providers’ lack of information about the disease is one of the barriers to the delivery of care for patients with chronic renal failure (1). As a result, it is necessary to carry out some training intervention for patients and medical staffs (20).

Hemodialysis skill was one of the factors facilitating hemodialysis care for the patients. Patients are in need of nurses who have specific knowledge and skills (21). A nurse who works in the field of hemodialysis, should be not only a capable person, but also must be knowledgeable and have enough information about the disease, dialysis, patients and likely complications during hemodialysis; the nurses must also have technical skills combined with appropriate experience (22). Technical nursing skills, especially the nurses’ ability to venipuncture for hemodialysis patients, plays an important role in their comfort (19).

Experience was one of the factors facilitating care of hemodialysis patients. Hemodialysis is a process which is under the influence of technology, equipment, machines, needles, skills and knowledge. Nurses, before meeting the patients’ needs, require an understanding of technology, technical skills, knowledge, and experience (23).

One of the categories of this study was about the barriers to the delivery of care for patients undergoing hemodialysis. According to Bennett study, nurses may feel helpless when they face training barriers, and conflict in the clinical guidelines on hemodialysis. These challenges might be caused due to limitation of roles, guidelines, and regulations of the hospitals (17).

Shortage of nurses and heavy workload were one of the subcategories of barriers of care for hemodialysis patients. According to Penoyer study, most studies suggest that the reduction in the number of nursing staff can lead to adverse unwanted outcomes in patients (21). Lack of access to human resources, unregulated care systems, and excessive workload inhibit the delivery of continuous and comprehensive care for the patients (2).

The weak authority of the head nurse and the ignorant director of nursing were the other subcategories of barriers to care for hemodialysis patients. According to Prezerakos et al, the ability of director of nursing to design a framework for the delivery of high quality nursing care is one of the most important factors in the workplace (24). Accruing to the results of a study by Aiken et al, a total of 34000 nurses who worked in various hospitals in 12 European countries, reported that the most important barriers to care are inadequate staff, lack of resources, and weak human resources management (25). In a study by Bennett, nurses said that the quality of nursing care is under the influence of the head nurses, hospital or health center managers, and nephrologists; the head nurse and the manager of hospitals have the strongest impact on the quality of nursing care. According to the patients, the ward manager, nurses, and environmental factors could influence the quality of care for hemodialysis patients (17).

The shortage of nephrologists was one of the subcategories of barriers to care for hemodialysis patients. Patients are interested to be visited by a nephrologist, especially when they have complex medical problems (18). Participation of clinical nephrologist, together with education and research can provide high quality care for a larger number of patients (18).

The lack of vascular surgeon was one of the other subcategories of barriers to care for hemodialysis patients. It is of great importance to make collaboration among physicians from different field (nephrologists, primary care physician, cardiologist, endocrinologist, vascular surgeons, and transplant specialists), and it is also important to engage different caregivers (nurses, pharmacists, social workers, and nutritionists) (1). Moreover, due to the complex management of chronic renal failure and because of the shortages felt in this field, a multi-disciplinary care team is necessary to provide care for the patients (20).

The lack of nurse’s aide and nursing assistant, unskilled staffs, and interventions by caregivers were among the other subcategories of barriers to care for hemodialysis patients. According to Garbin and Chmielewski study, to provide safety and quality of care for patients on dialysis, nephrology nurses should fully understand the role of the health care team members, so that they would become able to let other people to take care of the patients (26). Caregivers are often considered as supportive individuals, but sometimes patients introduce them as a source of stress and discomfort (7).

## Conclusion

The findings of this study showed that access to human resources and their abilities were among the factors facilitating care; however, lack of qualified medical staff at each level of care delivery was one of the barriers to hemodialysis care. Hence, it is of great importance for policy makers, managers, and program designers to recruit human resources who have the characteristics and competencies required for providing hemodialysis care.

## Limitations of the study

The study had some limitations such as small sample size and short duration of follow-up, thus we recommend to conduct of similar studies as multi-centric with longer duration of follow-up.

## Acknowledgments

We would like to thank the Deputy of Research and Tech­nology of Semnan University of Medical Sciences who financially supported this project. Also we would like to thank the clinical research development unit of Kowsar educational, research and therapeutic center of Semnan University of Medical Sciences for providing facilities to this work. In addi­tion, we appreciate the patients, caregivers, nurses, physi­cians in hemodialysis intensive care unit and the head of health center in Kowsar hospital in Semnan University of Medical Sciences.

## Authors’ contribution

MN; study design, data gathering, data interpretation and preparation of manuscript; MRT: final revision.

## Conflicts of interest

The authors declared no competing interests.

## Ethical considerations

Ethical issues (including plagiarism, data fabrication, double publication) have been completely observed by the authors.

## Funding/Support

This paper was extracted from a research project registered under the number 621, April 13, 2014; it was also approved by the Ethics Committee of Semnan University of Medical Sciences (No. 420741/92 – March 15, 2014).

## References

[1] Rastogi A, Linden A, Nissenson AR (2008). Disease management in chronic kidney disease. Adv Chronic Kidney Dis.

[2] Tejada-Tayabas LM, Partida-Ponce KL, Hernandez-Ibarra LE (2015). Coordinated hospital-home care for kidney patients on hemodialysis from the perspective of nursing personnel. Rev Lat Am Enfermagem.

[3] Guerra-Guerrero V, Sanhueza-Alvarado O, Caceres-Espina M (2012). Quality of life in people with chronic hemodialysis: association with sociodemographic, medical-clinical and laboratory variables. Rev Lat Am Enfermagem.

[4] Oller GA, Ribeiro Rde C, Travagim DS, Batista MA, Marques S, Kusumota L (2012). Functional independence in patients with chronic kidney disease being treated with haemodialysis. Rev Lat Am Enfermagem.

[5] Griva K, Ng HJ, Loei J, Mooppil N, McBain H, Newman SP (2013). Managing treatment for end-stage renal disease-- a qualitative study exploring cultural perspectives on facilitators and barriers to treatment adherence. Psychol Health.

[6] Chenitz KB, Fernando M, Shea JA (2014). In-center hemodialysis attendance: patient perceptions of risks, barriers, and recommendations. Hemodial Int.

[7] Krespi MR, Bone M, Ahmad R, Worthington B, Salmon P (2008). [Hemodialysis patients’ evaluation of their lives]. Turk Psikiyatri Derg.

[8] Axelsson L, Randers I, Jacobson SH, Klang B (2012). Living with haemodialysis when nearing end of life. Scand J Caring Sci.

[9] Silva AS, Silveira RS, Fernandes GF, Lunardi VL, Backes VM (2011). [Perceptions and changes in the quality of life of patients submitted to hemodyalisis]. Rev Bras Enferm.

[10] Graneheim UH, Lundman B (2004). Qualitative content analysis in nursing research: concepts, procedures and measures to achieve trustworthiness. Nurse Educ Today.

[11] Polit D. Essentials of Nursing Research: Appraising Evidence for Nursing Practice. Philadelphia: Lippincott Williams & Wilkins; 2013.

[12] Streubert HJ, Rinaldi Carpenter D. Qualitative research in nursing: advancing the humanistic imperative. Philadelphia: Lippincott Williams & Wilkins; 2011.

[13] Walker RC, Howard K, Morton RL, Palmer SC, Marshall MR, Tong A (2016). Patient and caregiver values, beliefs and experiences when considering home dialysis as a treatment option: a semi-structured interview study. Nephrol Dial Transplant.

[14] Urzúa A, Pavlov R, Cortés R, Pino V (2011). Psychosocial factors linked to health related quality of life in hemodialysis patients. Terapia Psicológica.

[15] Orlandi Fde S, Pepino BG, Pavarini SC, Dos Santos DA, de Mendiondo MS (2012). The evaluation of the level of hope of elderly chronic kidney disease patients undergoing hemodialysis. Rev Esc Enferm USP.

[16] Rajeswari R. Nursing care in dialysis. Chennai: Dialysis Technician Government General Hospital, Chennai; 2010.

[17] Bennett PN (2011). Satellite dialysis nursing: technology, caring and power. J Adv Nurs.

[18] Harwood L, Wilson B, Heidenheim AP, Lindsay RM (2004). The advanced practice nurse-nephrologist care model: effect on patient outcomes and hemodialysis unit team satisfaction. Hemodial Int.

[19] Borzou SR, Anoosheh M, Mohammadi E, Kazemnejad A (2014). Exploring perception and experience of patients from nursing care behaviors for providing comfort during hemodialysis. Journal of Qualitative Research in Health Sciences.

[20] Junaid Nazar CM, Kindratt TB, Ahmad SM, Ahmed M, Anderson J (2014). Barriers to the successful practice of chronic kidney diseases at the primary health care level; a systematic review. J Renal Inj Prev.

[21] Penoyer DA (2010). Nurse staffing and patient outcomes in critical care: a concise review. Crit Care Mede.

[22] Group Writers. Dialysis Nurse. 2nd ed. Tehran: Ministry of Health and Medical Education; 2009.

[23] Bennett PN (2011). Technological intimacy in haemodialysis nursing. Nurs Inq.

[24] Prezerakos P, Galanis P, Moisoglou I (2015). The work environment of haemodialysis nurses and its impact on patients’ outcomes. Int J Nurs Pract.

[25] Aiken LH, Sloane DM, Bruyneel L, Van den Heede K, Sermeus W (2013). Nurses’ reports of working conditions and hospital quality of care in 12 countries in Europe. Int J Nurs Stud.

[26] Garbin MG, Chmielewski CM (2013). Job analysis and role delineation: LPN/LVNs and hemodialysis technicians. Nephrol Nurs J.

